# Virtual reality in shoulder rehabilitation and shoulder arthroplasty pathways: a narrative review of current evidence and clinical perspectives

**DOI:** 10.1007/s00296-026-06200-6

**Published:** 2026-06-17

**Authors:** Anna Lach-Gruba, Paweł Turczyn, Stanisław Brodacki, Ewa Cieślicka, Anna Karwowska, Beata Żuk, Maria Maślińska, Dariusz Szukiewicz, Piotr Wojdasiewicz

**Affiliations:** 1 Department of Rehabilitation, St. Anna’s Hospital, Mazovian Rehabilitation Center—STOCER, Barska 16/20, Warsaw, 02-315 Poland; 2 Department of Early Arthritis, Rheumatology and Rehabilitation, Eleonora Reicher National Institute of Geriatrics, Spartańska 1, Warsaw, 02-637 Poland; 3 Department of Biophysics, Physiology and Pathophysiology, Faculty of Health Sciences, Medical University of Warsaw, 5 Chałubińskiego Street, Warsaw, 02-004 Poland; 4 Department of Rheumatology, Rheumatology and Rehabilitation, Eleonora Reicher National Institute of Geriatrics, Spartańska 1, Warsaw, 02-637 Poland

**Keywords:** Virtual reality, Shoulder rehabilitation, Shoulder arthroplasty, Upper limb, Telerehabilitation, Physical and rehabilitation medicine, Digital health

## Abstract

Shoulder rehabilitation is prolonged, feedback-dependent, and frequently limited by adherence, particularly after surgery. Virtual reality (VR) and related immersive or semi-immersive technologies may support rehabilitation by combining motion tracking, visual feedback, graded repetition, and gamified engagement. This narrative review summarizes current evidence on VR and digitally assisted shoulder and upper-limb rehabilitation and critically evaluates the extent to which these data can inform post-arthroplasty pathways. A narrative review was performed using PubMed/MEDLINE, PubMed Central, and targeted cross-checking in Scopus, Web of Science, and the Directory of Open Access Journals up to May 2026. Search domains combined shoulder and upper-limb terms, virtual reality, augmented reality, mixed reality, extended reality, and exergaming terms, rehabilitation and telerehabilitation terms, and postoperative shoulder surgery or arthroplasty terms. Priority was given to systematic reviews, randomized or controlled studies, validation studies, feasibility studies, and clinician-perspective studies relevant to shoulder biomechanics and rehabilitation implementation. The available literature supports three main conclusions. First, consumer-grade immersive systems can provide reliable within-system shoulder motion monitoring, although absolute agreement across devices remains imperfect. Second, VR, exergaming, and digitally assisted rehabilitation have shown feasibility, high acceptability, and potential benefits for adherence, pain, range of motion, and patient-reported function in rotator cuff repair, adhesive capsulitis, subacromial impingement, and other shoulder disorders. Third, evidence directly specific to anatomic or reverse shoulder arthroplasty rehabilitation remains limited; therefore, extrapolation from rotator cuff repair, conservative shoulder disorders, and digital home-based arthroplasty rehabilitation should be made cautiously. Rehabilitation clinicians support supervised or hybrid use rather than autonomous unsupervised replacement of conventional care. VR should be interpreted as an adjunct to clinician-led rehabilitation, not as a stand-alone substitute. Its most plausible current roles are improving engagement, enabling structured repetition, supporting within-system range-of-motion monitoring, and extending supervised practice into home settings. Future studies should test procedure-specific, phase-based VR protocols for anatomic and reverse shoulder arthroplasty, with explicit attention to compensation control, safety limits, long-term outcomes, cost-effectiveness, and multidisciplinary oversight.

## Introduction

Shoulder pain constitutes one of the most frequent musculoskeletal disorders and remains a major reason for seeking medical advice across primary and specialized care settings [1–4]. The clinical presentation is heterogeneous, encompassing impingement syndromes, rotator cuff tears, adhesive capsulitis, glenohumeral arthropathy, postoperative stiffness, and sequelae of trauma or inflammatory diseases. Irrespective of etiology, shoulder pain produces substantial disability by limiting self-care, professional activity, and sleep, often leading to secondary deconditioning and chronic pain behavior [1–4].

In parallel with the ageing population and broader surgical indications, shoulder arthroplasty has experienced exponential growth, both in anatomic and reverse designs [5–7]. Registry data from the United States and Europe confirm annual increases exceeding 10%, establishing shoulder replacement as one of the fastest-expanding reconstructive procedures in orthopedics. Advances in implant geometry and biomechanics have yielded high rates of pain relief, but the long-term functional success of these operations depends heavily on rehabilitation quality [8–10]. Rehabilitation is not a passive phase that follows surgery; it is a dynamic continuation of clinical treatment, essential for restoring coordinated motion, strength, and proprioceptive control once structural integrity has been re-established.

Traditional rehabilitation protocols for shoulder disorders and postoperative recovery are recognized for their intensity and duration. They often require weeks or months of repetitive exercise, guided either in outpatient sessions or through structured home programs. The principle of gradual load progression must be balanced with the prevention of compensatory motion and overuse. Yet adherence to these programs remains a well-known weakness, frequently reported at below 60% in independent home phases. Diminished motivation, limited supervision, and lack of feedback contribute to suboptimal compliance, thereby compromising recovery. This characteristic of shoulder rehabilitation—repetitive, feedback-dependent, and long-term—makes it uniquely suitable for digitally supported and interactive solutions.

**Fig. 1:**
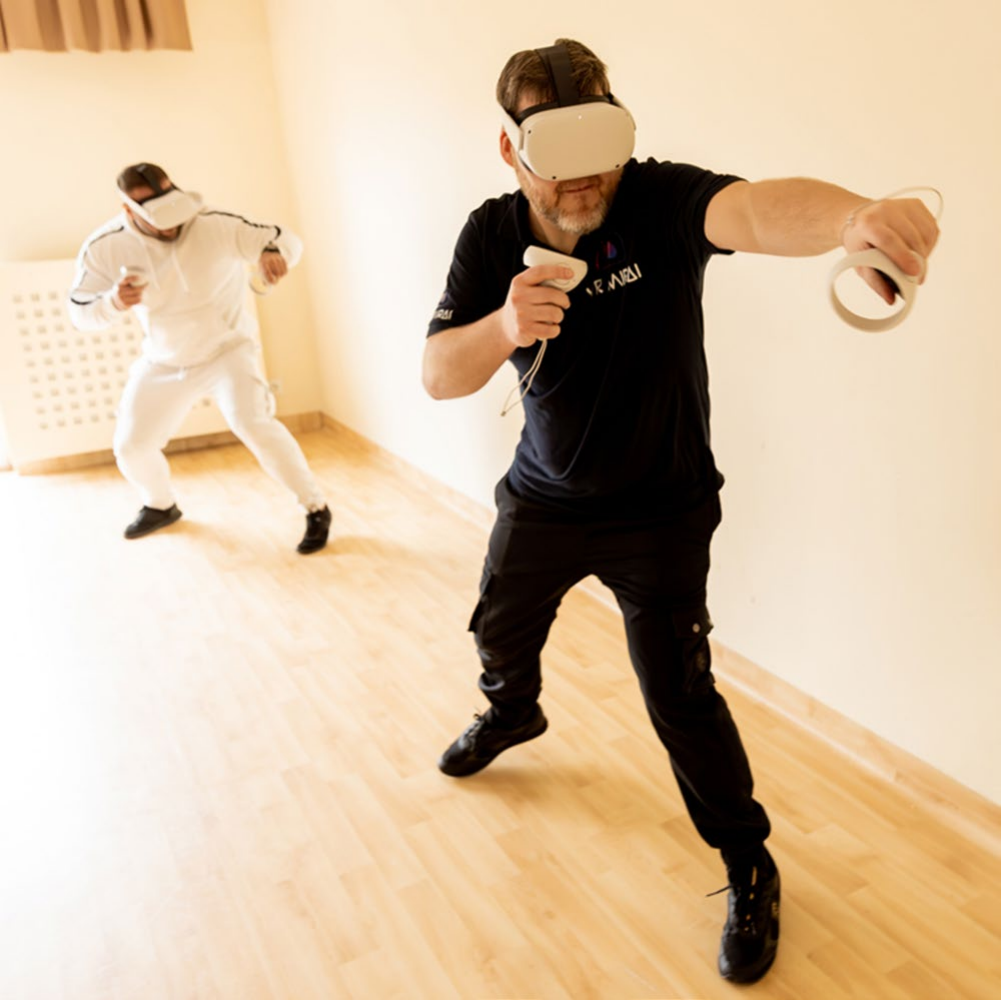
Example of immersive virtual reality–based upper-limb motor training using a head-mounted display and hand-held controllers. The boxing-inspired task illustrates the use of gamified repetitive movements in shoulder and upper-limb rehabilitation

Among available technologies, virtual reality (VR) is particularly compelling because it unifies exercise performance, motion quantification, and engagement within a single environment [11–16]. In its immersive form, VR surrounds the patient with a three-dimensional, computer-generated world in which real-world movements directly translate into controlled visual experiences. This interaction allows continuous feedback on motion amplitude, direction, and speed, while gamification elements strengthen motivation and reinforce adherence. An illustrative example of gamified VR-based upper-limb motor training is presented in Fig. 1. The feedback loop between sensory perception and motor execution embodies key mechanisms of neuroplastic learning—feedback, reward, and repetition—known to drive motor recovery.

Immersive systems have already demonstrated value in neurological, orthopedic, and pain rehabilitation by combining cognitive engagement with neuromuscular activation [12–15]. In selected rheumatologic contexts, particularly fibromyalgia, rheumatoid arthritis, chronic pain, and fatigue, VR has also been explored as a supportive strategy for symptom management, graded activity, relaxation, and motivation. These applications are relevant to shoulder rehabilitation only insofar as they address overlapping barriers such as fear of movement, reduced confidence, and long-term adherence; they do not by themselves establish procedure-specific effectiveness for shoulder arthroplasty.

Despite promising conceptual alignment, the field of VR-based shoulder rehabilitation remains heterogeneous and clinically uneven. Evidence includes technical validation, usability work, feasibility studies, small randomized trials, and digital telerehabilitation studies; however, most data concern rotator cuff repair or non-surgical shoulder conditions rather than shoulder arthroplasty itself [16–20]. Therefore, the present narrative review synthesizes current knowledge on VR-supported rehabilitation of the shoulder and upper limb, explicitly distinguishing direct arthroplasty-specific evidence from indirect evidence derived from related postoperative and conservative shoulder populations. It also examines the clinician-facing issues of terminology, supervision, safety, compensation control, and integration into anatomic and reverse shoulder arthroplasty pathways.

## Materials and methods

This review was designed as a narrative synthesis aimed at consolidating both technical and clinical evidence concerning VR application in shoulder rehabilitation. A narrative approach was considered appropriate given the methodological diversity, varying technological maturity, and early translational stage of the research.

### Search strategy and data sources

A systematic electronic search was carried out in PubMed/MEDLINE and PubMed Central and updated to May 2026. The search strategy was verified through targeted cross-checking in Scopus, Web of Science, and the Directory of Open Access Journals (DOAJ). The following representative search strings were applied, with database-specific adaptations where required: (1) (shoulder OR glenohumeral OR “upper limb” OR “upper extremity”) AND (“virtual reality” OR “augmented reality” OR “mixed reality” OR “extended reality” OR exergaming) AND (rehabilitation OR physiotherapy OR “physical therapy” OR telerehabilitation); (2) (“shoulder arthroplasty” OR “total shoulder arthroplasty” OR “reverse shoulder arthroplasty” OR “shoulder replacement”) AND (“virtual reality” OR “augmented reality” OR “digital rehabilitation” OR telerehabilitation); (3) (“rotator cuff repair” OR “rotator cuff tear”) AND (“virtual reality” OR exergaming OR “digital rehabilitation” OR “video-assisted” OR telerehabilitation); and (4) (fibromyalgia OR “rheumatoid arthritis” OR “chronic pain” OR fatigue) AND (“virtual reality” OR “VR meditation” OR “VR-based rehabilitation”). Manual screening of reference lists from relevant articles was also performed.

No language restrictions were initially applied; however, because the review required extractable methodological and clinical details, priority was given to peer-reviewed articles available in English. Titles and abstracts were screened independently by two reviewers, with discrepancies resolved by consensus. When evidence was inconsistent, greater interpretive weight was assigned to systematic reviews, randomized or controlled trials, prospective validation studies, and studies with clinically relevant shoulder-specific outcomes; pilot, developmental, and qualitative studies were used mainly to inform feasibility, usability, and implementation issues.

### Eligibility criteria and scope

Studies were included if they addressed at least one of the following thematic areas:

- Technical validation of VR platforms for shoulder or upper-limb motion assessment, including accuracy, reliability, or calibration studies;
- Clinical feasibility or application of VR-based rehabilitation in patients with shoulder disorders or postoperative conditions;
- Clinician perspectives, including studies of physiotherapists, rehabilitation physicians, orthopedic surgeons, or multidisciplinary teams addressing usability, safety, therapeutic acceptance, and implementation barriers;
- Postoperative or arthroplasty-specific rehabilitation using digital or VR-integrated technology.

Publications focusing exclusively on neurological recovery (e.g., stroke or spinal cord injury) were excluded unless they contained transferable insights on upper-limb biomechanics or task design relevant to shoulder movement. Grey literature, non-peer-reviewed proceedings, and off-topic case reports were omitted.

### Data extraction and synthesis

Information extracted from eligible papers included authorship, year, population, diagnosis or procedure, study design, VR or digital system used, immersion level (immersive virtual reality, semi-immersive exergaming, augmented reality, mixed reality, extended reality, or broader digital telerehabilitation), supervision model, comparator, outcome measures, follow-up duration, and main findings. Methodological characteristics were summarized descriptively, and a narrative methodological appraisal was assigned as lower, moderate, or higher concern based on study design, sample size, comparator, follow-up, and risk of confounding.

Because of heterogeneity in technology, intervention dose, supervision, and outcomes—ranging from motion accuracy and patient satisfaction to validated clinical measures such as the Shoulder Pain and Disability Index (SPADI), Disabilities of the Arm, Shoulder and Hand (DASH), Simple Shoulder Test (SST), range of motion (ROM), and visual analogue scale (VAS) pain scores—statistical pooling was not appropriate within this narrative review. Instead, a qualitative synthesis was undertaken, emphasizing research patterns, feasibility, therapist and clinician acceptance, indirectness of evidence, and implications for shoulder arthroplasty rehabilitation.

Inter-study consistency was evaluated narratively by comparing measurement outcomes, effect directions, usability trends, and the degree to which each study could reasonably inform shoulder arthroplasty pathways. Evidence was categorized as direct arthroplasty-specific evidence, indirect postoperative shoulder evidence, indirect conservative shoulder evidence, or contextual rheumatologic/chronic pain evidence.

### Terminology and evidence categories

For clarity, VR was defined as a computer-generated interactive environment presented through either immersive head-mounted displays or non-/semi-immersive screens and sensor-based interfaces. AR was defined as the overlay of digital information onto the real-world field, MR as interaction between digital and real-world elements, and XR as an umbrella term covering VR, AR, and MR. Exergaming and app-based telerehabilitation were considered separately from strictly immersive VR because several clinically relevant shoulder studies use digital feedback without fully immersive environments.

Throughout the review, evidence is therefore interpreted according to its proximity to the target clinical pathway. Studies after shoulder arthroplasty or explicitly modelling arthroplasty rehabilitation were considered direct or near-direct evidence. Studies after rotator cuff repair, adhesive capsulitis, impingement syndrome, and other non-arthroplasty shoulder conditions were considered indirect evidence. Rheumatologic and chronic pain studies were considered contextual and were used only to inform adherence, symptom modulation, and patient engagement.

## Evidence synthesis

Over the past decade, VR has evolved from an experimental adjunct into a credible candidate for inclusion in structured musculoskeletal rehabilitation. Within the upper-limb and shoulder domain, research has concentrated on three major trajectories: validating hardware and motion-capture algorithms, developing therapeutic applications tailored to shoulder biomechanics, and testing clinical usability or preliminary outcomes. These strands of evidence are promising but not equivalent: technical accuracy, patient engagement, and indirect postoperative findings should not be interpreted as proof of arthroplasty-specific clinical effectiveness.

For this reason, the synthesis below deliberately separates (i) technical validation, (ii) therapeutic applications in rotator cuff repair and conservative shoulder disorders, (iii) clinician perspectives and usability, (iv) comparative digital or VR-assisted rehabilitation trials, and (v) direct or near-direct evidence for shoulder arthroplasty pathways.

### Technical foundations and motion-capture accuracy

The accurate measurement of joint kinematics is an indispensable prerequisite for any clinically meaningful VR application. Traditional optical and inertial capture systems, while highly precise, are expensive and impractical for widespread therapeutic use. Therefore, early VR research aimed to evaluate whether consumer-grade head-mounted displays could achieve sufficient fidelity for clinical deployment. Carnevale et al. [21] rigorously quantified the translational and rotational accuracy of the Oculus Quest 2 headset. Using a nine-camera optoelectronic reference system as a gold standard, they demonstrated mean angular errors below 2°, confirming that consumer VR can reliably reproduce upper-limb trajectories during rehabilitation-like movement sequences. This validation bridged the gap between entertainment hardware and medical functionality.

Dejaco et al. [22] extended this validation to clinically relevant movements by examining flexion and scaption tasks in both healthy volunteers and postoperative patients. They compared VR-derived angles with smartphone inclinometry, obtaining correlation coefficients greater than 0.9, while noting a systematic overestimation of range of motion. The authors concluded that VR measurements are highly reliable for within-system tracking of progression, even though absolute comparability across devices is not yet achievable. These observations introduced an important conceptual nuance to digital outcome monitoring: consistency within a given technological ecosystem may be clinically sufficient if the system reliably measures change over time. Together, these studies provided the empirical foundation for using stand-alone headsets as low-cost motion laboratories. Importantly, both demonstrated the practicality of setting up immersive rehabilitation without external cameras or markers, a key step toward scalable home-based applications.

### From measurement to therapy: immersive applications

Following technical validation, attention turned to the design of interactive rehabilitation protocols adapted to the shoulder’s biomechanical complexity. Carnevale et al. [23] developed and piloted an immersive program specifically created for rehabilitation after arthroscopic rotator-cuff repair. The software simulated reaching and lifting tasks within a three-dimensional virtual environment, with visual feedback on speed, trajectory, and joint amplitude. Fifteen patients completed supervised VR sessions at defined postoperative intervals corresponding to the standard phase progression of cuff-repair rehabilitation. Quantitative analysis showed high adherence and accuracy in flexion and abduction tasks, reaching motion ranges close to those recommended for the respective protocol stage. However, internal and external rotations were associated with increased trunk compensation, as detected through auxiliary markers. This finding underscored the importance of implementing compensation-control algorithms or multimodal sensor input capable of distinguishing glenohumeral from scapulothoracic motion. Despite these limitations, patients reported high enjoyment and minimal fatigue, demonstrating the feasibility of combining therapeutic goals and motivational design.

Comparable concepts emerged in the management of non-surgical shoulder conditions. Pekyavas and Ergun [24] investigated an exergaming-based intervention for subacromial impingement syndrome and scapular dyskinesis. Participants were randomized to conventional home exercises or gaming-style interactive sessions guided by motion sensors. After six weeks, the VR-exergaming cohort achieved greater improvements in VAS pain, strength, and DASH score. The interactive component, as the authors emphasized, facilitated adherence far beyond that observed in unsupervised home programs.

Lee et al. [25] advanced the technical sophistication further by developing an inertial-sensor-based VR system capable of visualizing “motor ingredients” of shoulder motion. Real-time three-dimensional rendering enabled participants with adhesive capsulitis to monitor scapular rhythm and movement symmetry, resulting in measurable increases in total glenohumeral range and reduced fear of movement. Alvarez de la Campa Crespo et al. [26] added a neurocognitive layer by exploring virtual embodiment: patients controlling an avatar arm in an immersive environment experienced transient increases in pain-free motion, suggesting central modulation of body image and motor inhibition mechanisms. Collectively, these studies supplied preliminary physiological plausibility for VR as both a physical and perceptual rehabilitation tool.

### Usability and clinician perspectives

The adoption of new technology in rehabilitation practice depends equally on professional acceptance among rehabilitation clinicians, including physiotherapists, physical and rehabilitation medicine specialists, and orthopedic teams. Longo et al. [17] captured physiotherapists’ responses to immersive shoulder rehabilitation with Oculus Quest 2, applying the System Usability Scale and semi-structured feedback interviews. The mean usability score exceeded the threshold for “excellent,” with therapists describing the system as safe, ergonomically comfortable, and capable of maintaining patient interest through gamified tasks. Participants nevertheless expressed a clear preference for supervised sessions, particularly during early postoperative stages. Only 23% considered unsupervised home use acceptable.

Complementary qualitative studies by Brady et al. [18] and Dejaco et al. [19] illuminated nuanced professional attitudes. Therapists identified three perceived advantages: enhanced patient engagement, improved adherence through task variability, and objective documentation of movement data. However, they also highlighted barriers—setup cost, calibration time, and the absence of robust evidence demonstrating outcome superiority. The overarching theme across interviews was conditional optimism: VR is welcomed when positioned as an extension of therapist reasoning, not as its replacement.

These perspectives mirror historical patterns observed with earlier rehabilitation technologies such as biofeedback or robotic therapy, where eventual adoption followed proof of equivalence rather than promise of novelty. Importantly, they imply that future implementation success will depend not only on patient enthusiasm but on training, reimbursement models, and workflow compatibility within physiotherapy departments.

### Comparative clinical evidence and outcome assessment

A smaller but increasingly informative subset of research has examined VR, AR, exergaming, or digitally supported rehabilitation through quantitative outcome studies. Shim et al. [27] conducted a randomized controlled trial involving one hundred patients following rotator cuff repair; participants using a digital healthcare system with individualized exercise guidance and remote monitoring achieved greater improvements in SPADI and SST scores at twelve weeks compared with standard care. More recent syntheses of digital or VR-based rehabilitation after rotator cuff repair reported generally comparable or favorable outcomes for shoulder function, range of motion, and adherence, but also emphasized heterogeneity, limited numbers of randomized controlled trials (RCTs), and uncertainty regarding long-term superiority [28, 29].

Similarly, Correia et al. [30] performed a multicenter trial comparing digitally assisted home-based programs with conventional rehabilitation. Their results demonstrated non-inferiority in strength and range-of-motion outcomes, with a 35% reduction in therapist workload and high patient satisfaction. Kanat et al. [31] reported that video-assisted training after rotator-cuff repair significantly improved functional capacity compared with routine exercise, emphasizing the value of visual feedback even outside immersive environments. Menek et al. [32] confirmed comparable tendencies in chronic rotator-cuff rupture, where both video-gaming-based and closed-kinetic-chain programs outperformed traditional physiotherapy in targeted motor outcomes. Recent randomized trials in frozen shoulder further support VR-based exercise or immersive VR exergaming as adjunctive approaches for pain, range of motion, quality of life, and patient-reported outcomes, although these data remain indirect for arthroplasty pathways [33, 34]. Collectively, these results support the proposition that digital or semi-immersive feedback systems augment the therapeutic effect of conventional rehabilitation.

Despite methodological heterogeneity, three overarching trends can be distilled from these findings. First, immersive and semi-immersive technology reliably enhances motivation and adherence, translating into higher session completion rates. Second, accessibility through home connectivity enables scalable, partial decentralization of care. Third, the cost-to-benefit relationship remains favorable in pilot analyses because therapist time can be redirected toward evaluation rather than rote supervision.

### Direct and indirect evidence for postoperative shoulder arthroplasty pathways

Direct clinical evidence concerning immersive VR rehabilitation after anatomic or reverse shoulder arthroplasty remains sparse. Nam et al. [20] presented one of the earliest demonstrations of procedure-specific modelling, designing individualized VR pathways for arthroplasty, cuff repair, and stabilization surgery. Their prototype integrated surgery-specific movement restrictions and gradually expanded range-of-motion limits according to biologically informed healing phases. Although this was a technical feasibility report rather than an outcome trial, it illustrates how VR could serve as a digital scaffold for phase-based recovery.

Near-direct and indirect evidence comes from two related sources. First, web-based or home-based rehabilitation studies after shoulder arthroplasty suggest that structured remote rehabilitation can be feasible under appropriate monitoring [35–37]. Second, RCTs and meta-analyses after rotator cuff repair support the broader principle that digital feedback and remote monitoring may achieve comparable or sometimes improved functional outcomes compared with conventional programs [27–30]. These data support feasibility and plausibility, but they should not be presented as definitive proof of VR effectiveness after shoulder arthroplasty. A recent systematic review focused on metaverse, VR, and AR applications in total shoulder arthroplasty similarly indicates that this field remains early, heterogeneous, and insufficiently supported by procedure-specific clinical outcome trials [38].

The elective nature of shoulder arthroplasty and its predictable phase-based recovery make it suitable for structured digital support, but safety boundaries differ between procedures. After anatomic total shoulder arthroplasty, early VR modules should respect soft-tissue repair, subscapularis precautions where relevant, and gradual passive-to-active progression. After reverse shoulder arthroplasty, the program should prioritize deltoid and scapulothoracic control, avoid unsafe combined positions, and detect trunk or scapular substitution. Accordingly, VR should currently be framed as a supervised adjunctive extension of standard care, not as an independent arthroplasty protocol. The main characteristics, methodological profiles, and key findings of the studies discussed in this evidence synthesis are summarized in Table 1.

**Table 1:** Evidence map of VR-based, exergaming, and digitally assisted shoulder rehabilitation studies, with attention to population, supervision, comparator, follow-up, and methodological concern

Study	Population / diagnosis or procedure	VR or digital modality	Supervision and comparator	Outcomes and follow-up	Methodological concern*	Relevance for arthroplasty pathway
Carnevale et al. 2022 [21]	Healthy volunteers; technical validation	Immersive Oculus Quest 2 motion tracking	Compared with optical motion capture	Angular/translational accuracy; laboratory assessment	Lower for technical validation	Supports within-system motion monitoring but not clinical effectiveness
Dejaco et al. 2023 [22]	Healthy volunteers and shoulder patients; ROM measurement	Immersive VR measurement	Compared with smartphone inclinometry	Flexion/scaption ROM; reliability/validity	Lower to moderate	Useful for tracking progression; absolute device agreement remains imperfect
Carnevale et al. 2023 [23]	Post-rotator cuff repair; *n* = 15	Immersive VR rehabilitation application	Supervised feasibility; no conventional comparator	Task completion, ROM targets, compensation; postoperative phases	Higher due to small uncontrolled design	Important warning that virtual task success may mask trunk/scapular compensation
Longo et al. 2023 [17]	Physiotherapists evaluating shoulder VR program; *n* = 22	Immersive Oculus Quest 2 program	Professional usability assessment; no patient outcome comparator	System Usability Scale and interviews	Moderate	Supports supervised adoption and identifies reluctance toward unsupervised home use
Brady et al. 2023 [18]; Dejaco et al. 2024 [19]	Physiotherapist focus groups	VR-supported shoulder rehabilitation concepts	Qualitative professional perspectives	Perceived benefits, barriers, workflow issues	Moderate	Highlights clinician reasoning, training, workflow, and reimbursement as implementation determinants
Shim et al. 2023 [27]	Post-rotator cuff repair; RCT *n* = 100	Digital/AR-based rehabilitation and remote monitoring	Digital system vs. standard care	SPADI, DASH, SST, EQ-5D-5 L; 12 weeks	Moderate	Indirect postoperative evidence supporting digital feedback and monitoring
Correia et al. 2022 [30]	Post-arthroscopic rotator cuff repair; multicenter RCT *n* = 90	Home-based digital rehabilitation platform	Remote digital program vs. conventional rehabilitation	ROM, strength, satisfaction, therapist workload; short-term follow-up	Moderate	Suggests home digital care can reduce workload without loss of outcomes
AlHossan et al. 2025 [28]	Rotator cuff tears and post-repair recovery; systematic review/meta-analysis	Digital and VR-based rehabilitation	Compared with conventional therapy across included studies	Shoulder function, ROM, strength, pain, adherence	Moderate; heterogeneity and few RCTs	Supports plausibility but remains indirect for arthroplasty
Salimi et al. 2025 [29]	Post-rotator cuff repair; meta-analysis of RCTs	Digitally assisted home rehabilitation	Digital home program vs. conventional home-based rehabilitation	DASH and ROM outcomes; short-term trials	Moderate	Supports comparable outcomes in related postoperative shoulder rehabilitation
Pekyavas and Ergun 2017 [24]	Subacromial impingement syndrome and scapular dyskinesis; controlled trial *n* = 40	Sensor-based exergaming	Interactive sessions vs. home exercise	VAS, DASH, strength; 6 weeks	Moderate	Shows engagement and functional gains in non-surgical shoulder pathology
Lee et al. 2016 [25]	Frozen shoulder; development/feasibility	Wearable sensor-based VR with 3D feedback	No robust comparator	Movement symmetry, ROM, confidence	Higher	Suggests potential for movement-quality feedback
Demir et al. 2025 [33]	Frozen shoulder; RCT *n* = 36	VR-based exercise program	VR exercise vs. conventional treatment	VAS, SPADI, ROM, SF-36; 4 weeks	Moderate	Indirect conservative evidence for pain and quality-of-life effects
Bisadi et al. 2026 [34]	Primary frozen shoulder; RCT *n* = 54	Customised immersive VR exergaming	VR adjunct vs. conventional functional exercises	Passive ROM, DASH, VAS; post-intervention and 12-week follow-up	Moderate	Recent RCT; supports VR as adjunct, not replacement
Nam et al. 2024 [20]	Shoulder surgery modules including arthroplasty; developmental report	Procedure-specific VR modules	Technical feasibility; no clinical comparator	Design logic and phase-specific restrictions	Higher	Closest arthroplasty-specific VR rehabilitation model; requires clinical outcome testing
Davis et al. 2020 [35]; Eriksson et al. 2009 [36]; Schick et al. 2023 [37]	Anatomic and reverse shoulder arthroplasty rehabilitation	Web-based or home-based non-VR rehabilitation	Remote/home program vs. supervised outpatient care	Patient-reported function and postoperative outcomes	Moderate	Near-direct evidence that structured remote arthroplasty rehabilitation can be feasible

*Methodological concern is a narrative appraisal for this review, not a full formal Cochrane risk-of-bias judgement. It reflects study design, sample size, comparator, follow-up, and likely confounding

AR, augmented reality; DASH, Disabilities of the Arm, Shoulder and Hand; EQ-5D-5 L, EuroQol 5-Dimension 5-Level questionnaire; RCT, randomized controlled trial; ROM, range of motion; SF-36, 36-Item Short Form Health Survey; SPADI, Shoulder Pain and Disability Index; SST, Simple Shoulder Test; VAS, visual analogue scale; VR, virtual reality

### Selected rheumatologic contexts: fibromyalgia, rheumatoid arthritis, chronic pain, and fatigue

Although the present review focuses primarily on shoulder and upper-limb rehabilitation, selected rheumatologic contexts are relevant because pain, fatigue, fear of movement, reduced motivation, and long-term adherence problems frequently influence rehabilitation participation. In fibromyalgia, VR-based rehabilitation has been studied as an adjunctive approach for pain management, functional ability, physical conditioning, range of motion, and motivation. A recent systematic review and meta-analysis suggested potential benefits of VR-based interventions in fibromyalgia, but these findings should be interpreted as contextual support for symptom-modulating rehabilitation rather than evidence specific to shoulder arthroplasty [39].

A complementary perspective is provided by studies exploring VR meditation in rheumatoid arthritis. Dreesmann et al. reported that VR meditation for fatigue in persons with rheumatoid arthritis was feasible and acceptable in a mixed-methods pilot study, suggesting a possible supportive role for immersive relaxation and symptom self-management [40].

For the purposes of the present review, these rheumatologic and chronic pain data should be used cautiously. They support the broader rationale for engagement, pacing, relaxation, education, and feedback, but they do not demonstrate that VR improves outcomes after shoulder arthroplasty. Future protocols for patients with overlapping rheumatologic disease should therefore remain individualized and supervised.

## Clinical implications, safety considerations, and future directions

The accumulated evidence indicates that VR represents a credible adjunct to contemporary shoulder rehabilitation rather than a disruptive replacement for conventional practice. Its strongest current value lies in supporting repetition, visual feedback, motivation, and adherence. This interpretation is consistent with digital or semi-immersive programs after rotator cuff repair and conservative management of impingement or adhesive capsulitis, where improvements have been reported in pain, range of motion, adherence, and patient-reported function [24–34]. However, the same findings also underline the central limitation of the field: most evidence remains indirect for shoulder arthroplasty.

In selected rheumatologic or chronic pain contexts, the behavioral dimension of VR may also be relevant. Functional limitation is often shaped not only by local joint pathology but also by fatigue, pain sensitization, fear of movement, and reduced confidence. VR may help address these barriers by creating a controlled, engaging, and individually paced environment, but these observations should be framed as supportive and contextual rather than procedure-specific evidence for arthroplasty rehabilitation [39, 40].

Motion quality control is the decisive safety issue for translation into post-arthroplasty rehabilitation. Carnevale et al. [23] showed that patients can complete virtual tasks while using compensatory trunk or scapular strategies. After anatomic total shoulder arthroplasty, such compensation may obscure inadequate glenohumeral recovery and may encourage premature active loading or unsafe external rotation depending on soft-tissue repair. After reverse shoulder arthroplasty, uncontrolled trunk substitution and excessive scapular hiking may undermine deltoid/scapulothoracic retraining and expose patients to unsafe combined positions during early recovery. Therefore, VR systems intended for arthroplasty pathways should include compensation detection, safe range limits, fatigue monitoring, and phase-specific progression rules.

“Supervised integration” should be defined operationally. In early postoperative phases, VR should be introduced only after clinical clearance and should use restricted tasks aligned with the surgeon-approved rehabilitation protocol. The patient should receive initial in-person instruction, periodic assessment by a physiotherapist or rehabilitation physician, and review of movement-quality data. Home-based VR may then be used as a structured extension of therapy, with automated alerts or clinician review when pain, range limits, excessive repetition, or compensatory patterns occur. This hybrid model preserves clinical reasoning while increasing opportunities for safe practice.

Several limitations temper current optimism. Most available studies involve small samples, short intervention periods, heterogeneous hardware/software configurations, and variable definitions of VR, AR, exergaming, and telerehabilitation. Outcome measures differ widely, and only a small number of trials extend beyond three months. Importantly, clinical VR outcome trials after shoulder arthroplasty are still lacking. Existing data confirm feasibility, acceptability, and plausibility, but they do not yet prove superiority over established rehabilitation or define implant- or procedure-specific safety outcomes.

Despite these constraints, three practical insights emerge. First, VR and related digital systems may improve adherence and engagement, which are clinically meaningful in prolonged shoulder rehabilitation. Second, rehabilitation clinicians generally support VR when it assists rather than replaces clinical decision-making. Third, digital connectivity may reduce travel and therapist workload while maintaining contact with the patient, provided that access, digital literacy, data privacy, and reimbursement barriers are addressed [17–19, 27–30].

Future arthroplasty-specific pathways should be phase-based. Early modules should emphasize education, pain-limited protected motion, and avoidance of unsafe ranges. Intermediate modules should support active motion while identifying scapular or trunk substitution. Later modules may target strengthening, endurance, and simulated activities of daily living. For anatomic arthroplasty, subscapularis protection and rotator cuff function are central. For reverse arthroplasty, deltoid conditioning, scapulothoracic coordination, and avoidance of unstable positions are more relevant. These distinctions should be embedded in any future VR protocol rather than treated as generic shoulder exercises.

Overall, the case for VR in shoulder rehabilitation is supported by convergent trends rather than definitive arthroplasty trials. Technical reliability enables credible motion tracking, immersive design fosters motivation, clinician engagement preserves safety, and digital connectivity extends care beyond the clinic. The decisive test will come from procedure-specific randomized studies in postoperative arthroplasty cohorts that include pain, function, adherence, compensation control, adverse events, cost-effectiveness, and long-term durability. Until such evidence is available, VR should be presented as a promising supervised adjunct within multimodal rehabilitation.

## Conclusions

VR and related digital rehabilitation systems are promising adjuncts for shoulder and upper-limb rehabilitation because they can combine repetition, feedback, motion monitoring, and patient engagement. The current evidence base is strongest for technical validation, usability, rotator cuff repair, and non-arthroplasty shoulder disorders; direct clinical evidence after anatomic or reverse shoulder arthroplasty remains limited. Therefore, translation into arthroplasty pathways should be cautious, supervised, phase-based, and procedure-specific. Future randomized trials should evaluate clinical effectiveness, safety, compensation control, cost, adherence, and long-term functional outcomes. Until then, VR should complement—not replace—the expertise of rehabilitation clinicians.

## Data Availability

Data sharing is not applicable to this article because no datasets were generated or analyzed during the current review.

## Declarations

### Generative AI and AI-assisted technologies in the writing process

The authors declare that no generative artificial intelligence or AI-assisted technologies were used in the preparation of this manuscript. All content was prepared, reviewed, and approved by the authors.

## References

[1] Lucas J, van Doorn P, Hegedus E, Lewis J, van der Windt D (2022) A systematic review of the global prevalence and incidence of shoulder pain. BMC Musculoskelet Disord 23:1073 36476476 10.1186/s12891-022-05973-8 PMC9730650

[2] Luime JJ, Koes BW, Hendriksen IJ, Burdorf A, Verhagen AP, Miedema HS, Verhaar JA (2004) Prevalence and incidence of shoulder pain in the general population: a systematic review. Ann Rheum Dis 63(4):407–413 10.1080/03009740310004667 15163107

[3] Kuijpers T, van der Windt DA, Boeke AJ, Twisk JW, Vergouwe Y, Bouter LM, van der Heijden GJ (2006) Costs of shoulder pain in primary care consulters: a prospective cohort study in The Netherlands. BMC Musculoskelet Disord 7:83 17078883 10.1186/1471-2474-7-83 PMC1635047

[4] Virta L, Joranger P, Brox JI, Eriksson R (2012) Costs of shoulder pain and resource use in primary health care: a cost-of-illness study in Sweden. BMC Musculoskelet Disord 13:17 22325050 10.1186/1471-2474-13-17 PMC3299609

[5] Best MJ, Aziz KT, Wilckens JH, McFarland EG, Srikumaran U (2021) Increasing incidence of primary reverse and anatomic total shoulder arthroplasty in the United States. J Shoulder Elb Surg 30(5):1159–1166 10.1016/j.jse.2020.08.010 32858194

[6] Harjula JNE, Paloneva J, Haapakoski J, Kukkonen J, Aarimaa V, Finnish Shoulder Arthroplasty Registry Group (2018) Increasing incidence of primary shoulder arthroplasty in Finland - a nationwide registry study. BMC Musculoskelet Disord 19(1):245 30031390 10.1186/s12891-018-2150-3 PMC6054850

[7] Rupani N, Combescure C, Silman A, Lubbeke A, Rees J (2024) International trends in shoulder replacement: a meta-analysis from 11 public joint registers. Acta Orthop 95:348–357 38888103 10.2340/17453674.2024.40948 PMC11184711

[8] Bullock GS, Garrigues GE, Ledbetter L, Kennedy J (2019) A systematic review of proposed rehabilitation guidelines following anatomic and reverse shoulder arthroplasty. J Orthop Sports Phys Ther 49(5):337–346 31021690 10.2519/jospt.2019.8616

[9] Brindisino F, Lorusso M, Usai M, Pellicciari L, Marruganti S, Salomon M (2023) Rehabilitation following shoulder arthroplasty: a survey of current clinical practice patterns of Italian physiotherapists. Arch Physiother 13(1):12 37277886 10.1186/s40945-023-00166-5 PMC10243052

[10] Howard MC, Trasolini NA, Waterman BR (2023) Optimizing outcomes after reverse total shoulder arthroplasty: rehabilitation, expected outcomes, and maximizing return to activities. Curr Rev Musculoskelet Med 16(4):145–153 36867393 10.1007/s12178-023-09823-5 PMC10043097

[11] Lohre R, Warner JJP, Athwal GS, Goel DP (2020) The evolution of virtual reality in shoulder and elbow surgery. JSES Int 4(2):215–223 32490405 10.1016/j.jseint.2020.02.005 PMC7256885

[12] Brady N, McVeigh JG, McCreesh K, Rio E, Dekkers T, Lewis JS (2021) Exploring the effectiveness of immersive virtual reality interventions in the management of musculoskeletal pain: a state-of-the-art review. Phys Ther Rev 26(4):262–275

[13] Gumaa M, Khaireldin A, Rehan Youssef A (2021) Validity and reliability of interactive virtual reality in assessing the musculoskeletal system: a systematic review. Curr Rev Musculoskelet Med 14(2):130–144 33512677 10.1007/s12178-021-09696-6 PMC7844107

[14] Tokgoz P, Stampa S, Wahnert D, Vordemvenne T, Dockweiler C (2022) Virtual reality in the rehabilitation of patients with injuries and diseases of upper extremities. Healthc (Basel) 10(6):1124 10.3390/healthcare10061124 PMC9222955 35742176

[15] Chaplin E, Karatzios C, Benaim C (2023) Clinical applications of virtual reality in musculoskeletal rehabilitation: a scoping review. Healthc (Basel) 11(24):3178 10.3390/healthcare11243178 PMC10742848 38132067

[16] Sassi M, Villa Corta M, Pisani MG, Nicodemi G, Schena E, Pecchia L, Longo UG (2024) Advanced home-based shoulder rehabilitation: a systematic review of remote monitoring devices and their therapeutic efficacy. Sens (Basel) 24(9):2936 10.3390/s24092936 PMC11086333 38733040

[17] Longo UG, Carnevale A, Andreoli F, Mannocchi I, Bravi M, Hadj Sassi MS, Santacaterina F, Carli M, Schena E, Papalia R (2023) Immersive virtual reality for shoulder rehabilitation: evaluation of a physical therapy program executed with Oculus Quest 2. BMC Musculoskelet Disord 24:859 37919702 10.1186/s12891-023-06861-5 PMC10621204

[18] Brady N, Dejaco B, Lewis J, McCreesh K, McVeigh JG (2023) Physiotherapist beliefs and perspectives on virtual reality supported rehabilitation for the management of musculoskeletal shoulder pain: a focus group study. PLoS ONE 18(4):e0284445 37058507 10.1371/journal.pone.0284445 PMC10104293

[19] Dejaco B, Brady N, Tankink A, Lewis J, van Goor H, Staal JB, Stolwijk N (2024) Experiences of physiotherapists considering virtual reality for shoulder rehabilitation: a focus group study. Digit Health 10:20552076241234738 38414562 10.1177/20552076241234738 PMC10898295

[20] Nam J, Koh YG, Chung S, Kim PS, Jang J, Park JH, Kang KT (2024) The application of virtual reality in shoulder surgery rehabilitation. Cureus 16(4):e58280 38752078 10.7759/cureus.58280 PMC11094526

[21] Carnevale A, Mannocchi I, Hadj Sassi MS, Carli M, De Luca G, Longo UG, Denaro V, Schena E (2022) Virtual reality for shoulder rehabilitation: accuracy evaluation of Oculus Quest 2. Sens (Basel) 22(15):5511 10.3390/s22155511 PMC9332705 35898015

[22] Dejaco B, de Jong LD, van Goor H, Staal JB, Stolwijk N, Lewis J (2023) The concurrent validity and reliability of virtual reality to measure shoulder flexion and scaption range of motion. Physiotherapy 120:95–102 37429093 10.1016/j.physio.2023.06.005

[23] Carnevale A, Mannocchi I, Schena E, Carli M, Hadj Sassi MS, Marino M, Longo UG (2023) Performance evaluation of an immersive virtual reality application for rehabilitation after arthroscopic rotator cuff repair. Bioeng (Basel) 10(11):1305 10.3390/bioengineering10111305 PMC10668954 38002429

[24] Pekyavas NO, Ergun N (2017) Comparison of virtual reality exergaming and home exercise programs in patients with subacromial impingement syndrome and scapular dyskinesis: short term effect. Acta Orthop Traumatol Turc 51(3):238–242 28446376 10.1016/j.aott.2017.03.008 PMC6197467

[25] Lee SH, Yeh SC, Chan RC, Chen S, Yang G, Zheng LR (2016) Motor ingredients derived from a wearable sensor-based virtual reality system for frozen shoulder rehabilitation. Biomed Res Int 2016:7075464 27642600 10.1155/2016/7075464 PMC5011756

[26] de la Alvarez M, Donegan T, Amestoy-Alonso B, Just A, Combalia A, Sanchez-Vives MV (2023) Virtual embodiment for improving range of motion in patients with movement-related shoulder pain: an experimental study. J Orthop Surg Res 18(1):729 37752613 10.1186/s13018-023-04158-w PMC10523655

[27] Shim GY, Kim EH, Baek YJ, Chang WK, Kim BR, Oh JH, Lee JI, Hwang JH, Lim JY (2023) A randomized controlled trial of postoperative rehabilitation using digital healthcare system after rotator cuff repair. NPJ Digit Med 6(1):95 37221303 10.1038/s41746-023-00842-7 PMC10204020

[28] AlHossan AM, Jahhaf RH, Alharbi AS, Alqahtani LM, Alshahrani RM, Alowaidah LT, Alshangiti HY, Degen RM (2025) Digital and virtual reality-based rehabilitation versus conventional therapy for rotator cuff tears and post-repair recovery: a systematic review and meta-analysis. JSES Rev Rep Tech 6(1):100584. 10.1016/j.xrrt.2025.09.003 41142763 10.1016/j.xrrt.2025.09.003 PMC12553048

[29] Salimi M, Keshtkar A, Mosalamiaghili S, Sharafatvaziri A, Feeley BT (2025) Digitally assisted vs conventional home-based rehabilitation after rotator cuff repair: A meta-analysis. World J Clin Cases 13(32):109464. 10.12998/wjcc.v13.i32.109464 41256352 10.12998/wjcc.v13.i32.109464 PMC12620810

[30] Correia FD, Molinos M, Luis S, Carvalho D, Carvalho C, Costa P, Seabra R, Francisco GE, Bento V, Alcobia A et al (2022) Digitally assisted versus conventional home-based rehabilitation after arthroscopic rotator cuff repair: a randomized controlled trial. Am J Phys Med Rehabil 101(3):237–249 33935152 10.1097/PHM.0000000000001780 PMC8826616

[31] Kanat C, Ugras GA, Unal R, Donmez SC, Tasdelen B, Oztuna FV (2024) The effect of video-assisted training on upper extremity problems and functions after rotator cuff repair: a randomized controlled trial. Turk J Med Sci 54(1):165–174 38812623 10.55730/1300-0144.5777 PMC11031163

[32] Menek B, Tarakci D, Tarakci E, Menek MY (2022) Investigation on the efficiency of the closed kinetic chain and video-based game exercise programs in the rotator cuff rupture: a randomized trial. Games Health J 11(5):298–306 35666235 10.1089/g4h.2021.0210

[33] Demir OB, Kablanoglu S, Sari PN, Alyanak B, Taskin Yilmaz F, Dursun E (2025) Effects of virtual reality exercise on pain, joint motion, and quality of life in patients with frozen shoulder: a randomized controlled study. Physiother Theory Pract 41(7):1435–1446. 10.1080/09593985.2024.2443027 39700302 10.1080/09593985.2024.2443027

[34] Bisadi A, Mahrooz MH, Heidari M, Majdoleslami B, Minaei R, Mehdipour S (2026 May) Evaluating the Efficacy of Virtual Reality Exergaming in Frozen Shoulder Rehabilitation: A Randomized Clinical Trial. Games Health J 9. 10.1177/2161783X261447613 10.1177/2161783X261447613 42104784

[35] Davis DE, Cox R, Patel MS, Lazarus M, Ramsey M, Namdari S (2020) Successful outcomes achieved via web-based, home program after total shoulder arthroplasty. Arch Bone Jt Surg 8(6):661–667 33313345 10.22038/abjs.2020.42832.2164 PMC7718566

[36] Eriksson L, Lindstrom B, Gard G, Lysholm J (2009) Physiotherapy at a distance: a controlled study of rehabilitation at home after a shoulder joint operation. J Telemed Telecare 15(5):215–220 19590025 10.1258/jtt.2009.081003

[37] Schick S, Elphingstone J, Paul K, He JK, Arguello A, Catoe B, Roberson T, Momaya A, Brabston E, Ponce B (2023) Home-based physical therapy results in similar outcomes to formal outpatient physical therapy after reverse total shoulder arthroplasty: a randomized controlled trial. J Shoulder Elb Surg 32(8):1555–1561 10.1016/j.jse.2023.03.023 37178958

[38] Longo UG, Lalli A, Gobbato B, Nazarian A (2024) Metaverse, virtual reality and augmented reality in total shoulder arthroplasty: a systematic review. BMC Musculoskelet Disord 25(1):396. 10.1186/s12891-024-07436-8 38773483 10.1186/s12891-024-07436-8 PMC11106997

[39] Brea-Gómez B, Pérez-Gisbert L, Fernández-Castro I, Valenza MC, Torres-Sánchez I (2025) Effects of Virtual Reality-Based Rehabilitation in the Treatment of Patients with Fibromyalgia Syndrome: A Systematic Review and Meta-Analysis of Randomized Clinical Trials. Games Health J 14(2):79–102. 10.1089/g4h.2023.0193 39907102 10.1089/g4h.2023.0193

[40] Dreesmann NJ, Buchanan D, Tang HJ, Furness Iii T, Thompson H (2023) Virtual Reality Meditation for Fatigue in Persons With Rheumatoid Arthritis: Mixed Methods Pilot Study. JMIR Form Res 7:e46209. 10.2196/46209 37847542 10.2196/46209 PMC10618887

